# Latent profile analysis and influence factors study of nurses’ job performance

**DOI:** 10.3389/fpsyg.2025.1474091

**Published:** 2025-01-30

**Authors:** Zhenfan Liu, Xiaoting Yan, Guifang Xie, Jing Lu, Zhitong Wang, Cui Chen, Jijun Wu, Wei Qing

**Affiliations:** ^1^Department of Nursing, Deyang People's Hospital, Deyang, China; ^2^Department of Nursing, Sichuan Nursing Vocational College, Chengdu, China; ^3^Department of Nursing, Mianzhu Second People's Hospital, Mianzhu, China

**Keywords:** latent profile analysis, nurses, job performance, perceived social support, occupational coping self-efficacy

## Abstract

**Objective:**

To investigate the current status of nurses’ job performance, to analyze the latent profile analysis of nurses’ job performance and their relationship with occupational coping self-efficacy, perceived social support and to explore the factors influencing the different profiles.

**Methods:**

From April to June 2024, 390 nurses from five general hospitals were facilitated to be selected as survey respondents using a general information questionnaire, job performance scale, occupational coping self-efficacy scale, and perceived social support scale. Latent profile analysis of nurses’ job performance were analyzed, and logistic regression was used to analyze the factors influencing different categories of nurses’ job performance.

**Results:**

390 nurses were finally included. Nurses job performance score was (45.05 ± 6.55). Nurses’ job performance could be categorized into three latent profile analysis: low job performance (13.9%), medium job performance (52.8%) and high job performance (33.3%). Logistic regression analysis showed that years of working experience, form of employment, perceived social support and occupational coping self-efficacy were the influencing factors of nurses’ job performance (all *p* < 0.05).

**Conclusion:**

Nurses’ job performance is moderate to high and heterogeneous. Nursing managers should focus on “low job performance” and “medium job performance” nurses, and intervene and support nurses according to the characteristics and influencing factors of the different categories in order to improve their performance.

## 1 Introduction

Currently, as the socio-economic level continues to rise and public health awareness increases, global aging will continue to intensify. According to the World Health Organization (WHO), the global older adult population in 2050 will be twice as large as that in 2015 (World Health Organization, 2022). China entered the aging society in 1999, and the proportion of older adult population is also as high as 18.7%. And the increase in the older adult population may directly lead to the increasing demand for healthcare. Adequate human resources for nurses, as an important part of health care, are essential to promote the health of the older adult population, cope with global demographic changes, and reduce the socio-economic burden (Cacchione, 2022). However, the shortage of nurses has been an important issue in the global healthcare industry (Tamata and Mohammadnezhad, 2023). At the same time, due to the fact that the status of nurses is not widely recognized by the society and the welfare benefits are not sufficiently guaranteed, more and more nurses are inclined to leave their jobs, resulting in a high turnover rate of more than 40% of nurses across the globe (Labrague et al., 2020; Li et al., 2024). This will not be conducive to maintaining the stability of the nursing workforce, coping with demographic changes, and safeguarding the health of society. Studies have reported that job performance can negatively affect turnover intentions, i.e., when nurses demonstrate positive job performance, their turnover intentions tend to be lower (Yesilyurt et al., 2021; Wang et al., 2023). Therefore, improving nurses’ job performance is important for reducing turnover intentions.

Job performance, as a concept in management science, mainly refers to a series of behaviors and results achieved by individuals in the process of accomplishing tasks, which mainly includes task performance and contextual performance (Wu et al., 2021). Nurses’ job performance refers to the results and contributions created by nurses using their professional knowledge and skills in clinical work (He et al., 2023). As an important monitoring index to measure the development level and service supply capacity of hospitals, nurses’ job performance has become a hot spot of research at home and abroad in recent years (Yu, 2022). It has been found (Song et al., 2024) that nurses with higher levels of job performance are able to provide patients with high-quality nursing services, which has a positive effect on promoting patients’ health. At the same time, higher levels of job performance can also directly reduce the turnover rate of nurses and alleviate the healthcare challenges posed by an aging population (Wang et al., 2023). Therefore, an in-depth study of nurses’ job performance to explore its underlying mechanisms and influencing factors is precisely one of the purposes of this study.

A large number of studies at home and abroad have found that nurses’ job performance is affected by many aspects, in addition to the role of general demographic information such as age, gender, literacy, economic income, etc., it is also affected by Perceived Organizational Support, Psychological safety, Transformational leadership, Job embeddedness, etc. (Hasan et al., 2023; Shao et al., 2022; Song et al., 2024). Perceived social support, as an important variable in positive psychology, mainly refers to an individual’s subjective perception and evaluation of available social resources (e.g., emotional support, information support, etc.) (Zimet et al., 1990). Specifically, when nurses feel more support, trust, and recognition from organizations, families, friends, etc., nurses are motivated to perform more positively at work. It has been pointed out that effective social support can alleviate the psychological burnout of nurses, thus showing positive work status; at the same time, nurses with higher levels of social support have less psychological stress and show better psychological capital, thus focusing on their own work (Huang et al., 2023; Wu et al., 2020;).

Nurses’ occupational coping self-efficacy refers to the subjective evaluation and awareness of nurses’ ability to effectively cope with and complete clinical nursing work, and is a positive judgment of their own behavioral competence and psychological state (Mei and Han, 2021). It has been found that nurses with high levels of occupational coping self-efficacy, as an intra-individual resource, are able to effectively cope with occupational stress, and thus are better engaged in their work and show higher levels of work productivity (Liu et al., 2023). Meanwhile, occupational coping self-efficacy reduces the intensity of transition shock and improves nurses’ ability to adapt to their careers, thus showing positive work status (Dai et al., 2023). In addition, according to the job demand-resource model (JD-R), when nurses have high levels of internal resources (e.g., occupational coping self-efficacy, psychological capital, etc.) and external resources (social support, organizational support, etc.), they can have positive organizational behaviors and show positive engagement in their work, which creates high levels of work outcomes (Topa and Aranda-Carmena, 2022). Therefore, it is reasonable to hypothesize that nurses’ level of perceived social support as well as occupational coping self-efficacy is strongly related to job performance.

However, previous studies on nurses’ job performance have mainly focused on status quo surveys and the exploration of the relationship between related variables, while whether there are heterogeneous differences within the nurse job performance group remains to be further analyzed (Aung Po et al., 2023). Meanwhile, there are few domestic and international studies on the relationship between nurses’ job performance and perceived social support and occupational coping self-efficacy, and the deeper mechanism of action between the variables is not clear. In addition, most of the studies have taken the form of scale total scores to judge the level of nurses’ job performance, ignoring the differences between individuals. Potential profiling is an individual-centered research method that accurately identifies potential categories between groups, which in turn enables a unique characterization of potential categories, enabling researchers to clearly understand the relationships between different types of individuals and clarify the nature and number of categories (Berlin et al., 2014).

Therefore, this study mainly used latent profile analysis to explore the latent profile analysis of nurses’ job performance, analyze the population characteristics and influencing factors among different profiles, and explore the relationship between job performance and perceived social support and occupational coping self-efficacy among different profiles, with a view to providing reference bases for managers to develop intervention methods for nurses’ job performance.

## 2 Methods

### 2.1 Objective

The purpose of this study was to explore the different subtypes of nurses’ job performance and to determine the relationship of each subtype to perceived social support, occupational coping self-efficacy, and related underlying factors.

### 2.2 Participants

This study conducted a cross-sectional survey from April to June 2024, and selected 390 nurses from five general hospitals as survey respondents through convenience sampling. Inclusion criteria: (1) having obtained a certificate of nursing practice and having worked in nursing for ≥3 months; (2) informed consent and willingness to participate in this study; Exclusion criteria: nurses on leave or in training during the survey period.

Sample size calculation: according to the potential profile analysis and Logistic regression analysis, the average sample size of each profile can be obtained when the average sample size of at least 50 stable statistical results, this study is proposed to explore the number of latent profile analysis ≥3, then the sample size of at least ≥150 cases. According to the empirical method of sample size calculation for Logistic regression analysis: the sample size is 10 to 15 times the number of independent variables, the number of independent variables is taken as 16, and the sample size is calculated as 160 to 240 cases (Ni et al., 2010). Considering 20% of invalid questionnaires, the minimum sample size of this study was 192 cases, and finally 390 samples were analyzed. The study was approved by the Ethics Committee (No. 2023–04-070-K01).

### 2.3 Methodology

#### 2.3.1 Survey instruments

##### 2.3.1.1 General information questionnaire

The general information questionnaire was determined by the research team after consulting relevant literature and includes items such as the nurse’s gender [1 = male; 2 = female], age, hospital level [1 = grade 3A; 2 = grade 3B; 3 = Level II and below], marital status [1 = unmarried; 2 = married], professional title [1 = junior ranking; 2 = middle level; 3 = high level], Forms of employment [1 = Staff nurse; 2 = Contract nurse], position [1 = not have; 2 = head nurse], highest level of education [1 = associate degree; 2 = bachelor’s degree; 3 = postgraduate and above], average monthly income,Number of weekly night shifts [1 = 0; 2 = 1; 3 = 2; 4 = 3 or more], weekly working hours [1 = 0 ~ 48 h; 2 = 41 ~ 48 h; 3 = more than 48 h], and years of work experience.

##### 2.3.1.2 Occupational coping self-efficacy scale

Compiled by Italian scholars (Pisanti et al., 2008) and revised by Chinese scholars (Zhai et al., 2021), the scale has high reliability and validity. The Cronbach′s *α* coefficient of the scale was 0.882. The Cronbach′s α coefficient for the survey measurement scale among nurses was 0.886 by Liu et al. (2022). The scale consists of 2 dimensions, difficulty getting along in relationships (3 items) and occupational burden (6 items), with a total of 9 items. The scale was scored on a 5-point Likert scale, with all positive scores ranging from 1 to 5, from “not able to cope easily” to “fully able to cope easily,” with a minimum of 9 points and a maximum of 45 points. The main purpose of this scale is to determine the level of nurses’ occupational coping self-efficacy, and the higher the total score, the higher the nurses’ occupational coping self-efficacy. In general, a mean score of <3 is considered to be a low level of performance, 3 ~ 4 is considered to be a medium level, and > 4 is considered to be a high level. The Cronbach′s *α* coefficient for this scale was 0.911 in this study.

##### 2.3.1.3 Perceived social support scale

Compiled by American scholars (Zimet et al., 1988) and revised by Chinese scholars (Zhong et al., 2005) in Chinese, the scale has high reliability and validity. The Cronbach′s *α* coefficient of the scale was 0.990. The Cronbach′s *α* coefficient for the survey measurement scale among nurses was 0.976 by He et al. (2023). The scale consists of three dimensions: family support (4 items), friend support (4 items), and other support (4 items), totaling 12 items (He et al., 2023). It uses a 7-point Likert scoring method, with all items scored positively, ranging from “strongly disagree” to “strongly agree” with values from 1 to 7. The minimum total score is 12, and the maximum is 84. This scale is primarily used to measure the level of perceived social support among nurses, with higher total scores indicating greater perceived social support. Generally, a total score < 36 is considered to represent low levels of social support, 37 ~ 60 represents moderate levels of social support, and > 60 represents high levels of social support. The Cronbach′s *α* coefficient for this scale was 0.967 in this study.

##### 2.3.1.4 Job performance scale

Compiled by American scholars Borman & Modowidlo and revised by Chinese scholar Yu De-cheng from Taiwan, the scale has high reliability and validity (Borman and Motowidlo, 1993; Yu, 1996). The Cronbach′s α coefficient of the scale was 0.918. The Cronbach′s *α* coefficient of the scale was 0.918 for the survey-determined scale applied to nurses by Chen et al. (2023). The scale includes two dimensions: task performance (6 items) and contextual performance (5 items), totaling 11 items. It uses a 5-point Likert scoring method, with all items scored positively, ranging from “completely disagree” to “completely agree” and assigned values from 1 to 5. The minimum total score is 11, and the maximum is 55. The scale is primarily used to measure the level of nurses’ job performance, with higher total scores indicating higher job performance. In general, a mean score of <3 is considered to be a low level of performance, 3 ~ 4 is considered to be a medium level, and > 4 is considered to be a high level. The Cronbach′s *α* coefficient for this scale was 0.940 in this study.

#### 2.3.2 Survey methods

This study was conducted in the form of the Chinese online platform Questionnaire Star (Wen Juan Xing,wjx.cn). In the first step, the project leader edited the questionnaire in accordance with the purpose and content of this project survey, including the purpose of the study, content, significance, filling out matters, informed consent, confidentiality of the questionnaire, and at the same time imported the questionnaire into the questionnaire star to create a link to conduct a pre-survey, and according to the results of the pre-survey to improve the questionnaire content to determine the final version; in the second step, the project leader contacted the relevant hospital questionnaire investigator, and after Second step, the project leader contacted the questionnaire investigators in the relevant hospitals, and after obtaining their informed consent, all the investigators were trained in the form of videoconferencing on the Tencent conferencing platform to carry out questionnaire investigations in a uniform standard, and after passing the training, the link and QR code of the questionnaire were sent to the research subjects; in the third step, the research subjects received the link or QR code, read the relevant content, and filled out the questionnaire entries in accordance with their own ideas under the circumstance of fully informed consent. To ensure the validity and completeness of the questionnaire, all questionnaire entries were required and each IP account had only 1 chance to answer. To ensure the confidentiality and ethical requirements of the research subjects’ results, firstly, the research subjects must be fully informed about the purpose, content, inclusion and exclusion criteria of the study and must consent to participate in the survey; secondly, the questionnaires do not require the subjects to provide their names or the names of their hospitals, and all research results are coded and accessible only to the research team members; finally, if the research subjects feel any discomfort during the survey process, they can decide at any time whether to withdraw from the investigation. A total of 402 questionnaires were collected, and after screening out the questionnaires with obvious errors in logic, and questionnaires with an answer time of <180 s or > 1800 s were regarded as invalid questionnaires, and 12 questionnaires were excluded, a total of 390 valid questionnaires were recovered, with an effective recovery rate of 97.0%.

#### 2.3.3 Statistical methods

The questionnaire results were directly exported from the background of Questionnaire Star. SPSS 25.0 software was used for statistical analysis. Measured data were expressed as mean ± standard deviation, and ANOVA was used for comparison between groups; count data were described as number and percentage, and chi-square test or rank-sum test was used for comparison between groups. Potential profile analysis of nurses’ job performance was performed using Mplus 8.3 software, starting with an initial model of 1 category, gradually increasing the number of model categories, and determining the optimal model according to the potential profile model evaluation indexes and conducting fitness tests and difference tests. The smaller the Akaike information criteria (AIC), Bayesian information criteria (BIC), and adjusted BIC (aBIC), the better the model fit; the entropy (entropy) is a measure of the accuracy of model classification is an evaluation index of model classification accuracy, taking the value of 0 ~ 1, the closer to 1 means the higher the accuracy; Lo-MendellRubin (LMR) and Bootstrap-based likelihood ratio (LMRT) are used to validate the differences in the model fit, and *p* < 0.05 indicates that the k-category model is better than the k-1-category model (Wang and Bi, 2018). Multinomial logistic regression analysis was performed with the classification results of latent profile analysis as the dependent variable and the variables that were statistically significant in the univariate analysis as the independent variables, with *p* < 0.05 indicating that the differences were statistically significant.

## 3 Results

### 3.1 General information on respondents

A total of 390 clinical nurses were included in this study, including 33 (8.5%) male nurses and 357 (91.5%) female nurses; age: 33.74 ± 7.35 years, and other general information is shown in Table 1.

**Table 1:** Single factor analysis of 3 latent profile analysis of nurse’s job performance [*n* = 390, case (percentage, %)].

Sports event		*n*	Low job performance (13.9%)	Medium job performance (52.8%)	High job performance (33.3%)	*χ^2^* value	*p*-value
Distinguishing between the sexes	Male	33	7 (13.0)	16 (7.8)	10 (7.7)	1.723	0.436
	Female	357	47 (87.0)	190 (92.2)	120 (92.3)		
Age group (years)	21–25	49	10 (18.5)	31 (15.0)	8 (12.6)	16.921	0.010
	26–30	99	19 (35.2)	47 (22.8)	33 (25.4)		
	31 to 40	178	23 (42.6)	94 (45.6)	61 (45.6)		
	>40	64	2 (3.7)	34 (16.6)	28 (16.4)		
Hospital level	Grade 3A	262	43 (79.6)	128 (62.1)	90 (69.2)	8.145	0.082
	Grade 3B	29	2 (3.7)	21 (10.2)	6 (4.6)		
	Level II and below	100	9 (16.7)	57 (27.7)	34 (26.2)		
Marital status	Unmarried	86	21 (38.9)	48 (23.3)	17 (13.1)	15.185	0.001
	Married	304	33 (61.1)	158 (76.7)	113 (86.9)		
Title	Junior ranking	196	35 (64.8)	101 (49.0)	60 (46.2)	6.508	0.160
	Middle level (in a hierarchy)	166	18 (33.3)	89 (43.2)	59 (45.4)		
	High level	28	1 (1.9)	16 (7.8)	11 (8.4)		
Forms of employment	Staff nurse	79	4 (7.4)	46 (22.3)	29 (22.3)	6.406	0.041
	Contract nurse	311	50 (92.6)	160 (77.7)	101 (77.7)		
Duties	Not have	326	51 (94.4)	162 (78.6)	113 (86.9)	9.369	0.009
	Head nurse	64	3 (5.6)	44 (21.4)	17 (13.1)		
Highest level of education	Associate degree	63	10 (18.5)	31 (15.0)	22 (16.9)	1.088	0.911
	Bachelor’s degree	308	41 (75.9)	166 (80.6)	101 (77.7)		
	Postgraduate and above	19	3 (5.6)	9 (4.4)	7 (5.4)		
Average monthly income of individuals	0 to 5,000	122	16 (29.6)	69 (33.5)	37 (28.5)	10.576	0.102
	5,001 to 7,500	136	18 (33.3)	72 (35.0)	46 (35.4)		
	7,501 to 10,000	86	18 (33.3)	43 (20.9)	25 (19.2)		
	10,001~	46	2 (3.8)	22 (10.6)	22 (16.9)		
Number of weekly night shifts	0	163	18 (33.3)	85 (41.3)	60 (46.2)	4.573	0.600
	1	49	6 (11.1)	25 (12.1)	18 (13.8)		
	2	96	18 (33.3)	51 (24.8)	27 (20.8)		
	3 or more	82	12 (22.3)	45 (21.8)	25 (19.2)		
Weekly working hours (h)	0 to 40	107	12 (22.2)	53 (25.7)	42 (32.3)	5.335	0.255
	41–48	186	27 (50.0)	95 (46.1)	64 (49.2)		
	>48	97	15 (27.8)	58 (28.2)	24 (18.5)		
Work experience band (years)	0 to 5 years	86	20 (37.0)	47 (22.8)	19 (14.6)	18.586	0.005
	6 to 10 years	109	15 (27.8)	60 (29.1)	34 (26.2)		
	11–20 years	143	18 (33.3)	71 (34.5)	54 (41.5)		
	>20 years	52	1 (1.9)	28 (13.6)	23 (17.7)		

### 3.2 Job performance, perceived social support, and occupational coping self-efficacy scores of survey respondents

The results of this study showed that clinical nurses scored (45.05 ± 6.55) for job performance, (70.25 ± 11.55) for perceived social support, and (37.22 ± 5.45) for occupational coping self-efficacy, and the scores of the dimensions are detailed in Table 2.

**Table 2:** Respondents’ scores on job performance, perceived social support, and occupational coping self-efficacy.

Sports event	*n*	Scoring range	Score
Occupational coping self-efficacy	9	9 to 45	37.22 ± 5.45
Difficulty getting along in relationships	3	3 to 15	12.77 ± 1.91
Professional burden	6	6 to 30	24.46 ± 3.81
Perceived social support	12	25 to 84	70.25 ± 11.55
Support from others	4	9–28	23.34 ± 3.95
Friends support	4	6–28	23.30 ± 4.15
Family support	4	4–28	23.61 ± 4.27
Job performance	11	21–55	45.05 ± 6.55
Contextual performance	5	10 to 25	20.80 ± 3.03
Task performance	6	10–30	24.26 ± 3.85

### 3.3 Latent profiles analysis of nurse performance

In this study, 11 entry scores of job performance were used as exogenous indexes, and 1 to 5 models were fitted sequentially, and the results of the fitting indices of each model are shown in Table 2. The results of the study showed that the *AIC*, *BIC*, and *aBIC* indices monotonically decreased with the increase in the number of profiles, and the Entropy values of categories 3, 4 and 5 were all >0.800, but the *LMR* indices of categories 4 and 5 were all >0.05 reached significance, indicating that the model may change as the categories increase, and after comprehensive consideration, model 3 was considered as the best profile model, and three profiles (C1, C2, C3) were retained. As can be seen from Table 3, the average probability of attribution for each profile was 98.6, 97.8, and 99.1% respectively, all above 90%, indicating that the results of the three potential profile models have credibility.

**Table 3:** Results of each fitted indicator of the potential profile analysis of nurses’ job performance.

Mould	*AIC*	*BIC*	*aBIC*	*LMR*	*BLRT-P*	*Entropy*	Profile scale
1	9677.213	9764.468	9694.664	-	-	-	-
2	7703.322	7838.171	7730.291	0.0001	0	0.965	0.605/0.395
3	6834.345	7016.787	6870.832	0.0046	0	0.968	0.528/0.139/0.333
4	6391.827	6621.863	6437.833	0.1438	0	0.978	0.451/0.113/0.297/0.139
5	5458.417	5736.047	5513.941	0.3044	0	0.978	0.085/0.087/0.277/0.408/0.143

**Figure 1:**
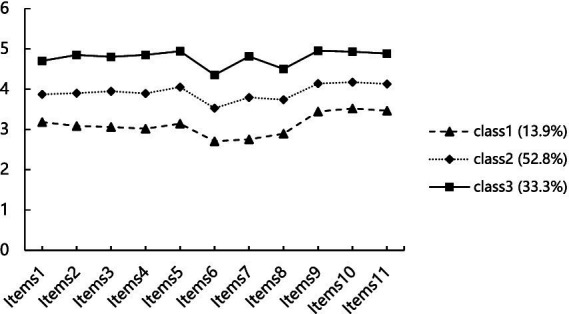
The characteristic distribution of 3 potential categories of nurse’s job performance.

Based on the classification results, the latent profiles of C1, C2, and C3 in job performance were plotted. There are no intersection points on each item for the three latent profiles, and the trends of the profile shapes are also consistent, indicating that there are differences among the three profiles, see Figure 1. The horizontal axis represents the 11 items of work performance, and the vertical axis represents their corresponding means. The higher the item score, the better the job performance of the nurse. At the same time, by combining the fluctuation of the scores of each item, the profiles are named. From the results of Figure 1, it can be seen that there are 54 nurses in profile C1 (13.9%), whose scores on each item are significantly lower than the other two groups and are at a lower level, indicating that the job performance created by this category of nurses is relatively low, hence named “Low job performance Type”; there are 206 nurses in profile C2 (52.8%), whose scores on each item are between the other two groups and are at a medium level, indicating that the job performance created by this category of nurses is relatively average, hence named “Medium job performance Type”; there are 130 nurses in profile C3 (33.3%), whose scores on each item are higher than the other two groups and are at a higher level, indicating that the job performance created by this category of nurses is relatively high, hence named “High job performance type.”

### 3.4 Univariate analysis of potential categories of nurses’ job performance

The results showed that the “low job performance” nurses scored (60.41 ± 9.38) in perceived social support, the “medium job performance” nurses scored (67.98 ± 10.08), and the “high job performance “nurses scored (77.95 ± 9.79). The differences among the three groups were statistically significant (*F* = 71.513, *p* < 0.001). The “low job performance” nurses scored (32.72 ± 5.09) in occupational coping self-efficacy, the “medium job performance” nurses scored (36.08 ± 4.25), and the “high job performance” nurses scored (40.90 ± 5.10). The differences among the three groups were statistically significant (*F* = 71.636, *p* < 0.001). A single-factor comparison of each dimension of the independent variables with different facets of job performance is shown in Table 4. The comparison of age, marital status, position, employment form, and years of work experience among different types of nursing job performance facets showed statistically significant differences (all *p* < 0.05). See Table 1.

**Table 4:** One-way analysis of potential categories of job performance of survey respondents with dependent variables.

Sports event	Low job performance	Medium job performance	High job performance	*F*	*P*
Occupational coping self-efficacy	32.72 ± 5.09	36.08 ± 4.25	40.90 ± 5.10	71.636	<0.001
Difficulty getting along in relationships	11.65 ± 1.94	12.37 ± 1.52	13.86 ± 1.97	42.199	<0.001
Professional burden	21.07 ± 3.53	23.71 ± 3.03	27.04 ± 3.44	75.969	<0.001
Perceived social support	60.41 ± 9.38	67.98 ± 10.08	77.95 ± 9.79	71.513	<0.001
Support from others	19.92 ± 3.12	22.52 ± 3.49	26.05 ± 3.24	75.631	<0.001
Friends Support	20.13 ± 3.57	22.43 ± 3.84	25.99 ± 3.25	62.556	<0.001
Family support	20.30 ± 3.90	23.03 ± 3.69	25.91 ± 4.08	45.327	<0.001

### 3.5 Multifactor logistic regression analysis of nurses’ job performance

The three categories of nurses’ job performance were used as dependent variables, and the variables that were meaningful in the univariate analysis were analyzed as independent variables in a multivariate logistic regression, and the independent variables were assigned the following values. Marital status: unmarried = 0, married = 1; form of employment: staffing = 0, non-staffing = 1; Duties: not have = 0, 1 = nurse head; age and years of experience were substituted with actual values, and occupational coping self-efficacy, and perceptual social support were substituted with actual scores on the scale. The results showed that the higher the scores on the Appreciative Social Support Scale, the higher the scores on the Occupational Coping Self-Efficacy Scale, the longer the years of service, and the more likely the non-established nurses were to enter the C2 group compared to the C2 group. The higher the scores on the Appreciative Social Support Scale, the higher the scores on the Occupational Coping Self-Efficacy Scale, and the more likely the non-established nurses were to enter the C3 group compared to the C1 group. Support scale scores, higher scores on the occupational coping self-efficacy scale, and nurses who were non-established were more likely to enter group C3 compared to group C2, see Table 5.

**Table 5:** Multivariate logistic regression analysis of influencing factors of nurse’s job performance.

Sports event	*B*	Standard error	Wald	*P*	OR	95% CI
C1 compared to C2^1^						
(A person’s) age	−0.143	0.080	3.158	0.076	0.867	0.741 ~ 1.015
Years of experience	0.149	0.076	3.862	0.049	1.161	1.000 to 1.347
Perceived social support	0.059	0.016	12.957	<0.001	1.060	1.027 ~ 1.095
Occupational coping self-efficacy	0.094	0.033	8.063	0.005	1.099	1.030 ~ 1.173
Marital status (unmarried)	−0.385	0.469	0.674	0.412	0.680	0.271 ~ 1.706
Form of employment (establishment)	1.600	0.702	5.194	0.023	4.954	1.251 ~ 19.614
Duties (none)	−0.569	0.707	0.649	0.421	0.566	0.142 ~ 2.262
C1 compared to C3^(1)^						
(A person’s) age	−0.070	0.094	0.553	0.457	0.932	0.775 ~ 1.121
Years of experience	0.115	0.088	1.688	0.194	1.122	0.943 ~ 1.334
Perceived social support	0.141	0.023	37.630	<0.001	1.152	1.101 to 1.205
Occupational coping self-efficacy	0.279	0.048	34.448	<0.001	1.322	1.204 ~ 1.451
Marital status (unmarried)	−0.773	0.591	1.709	0.191	0.462	0.145 ~ 1.471
Form of employment (establishment)	1.760	0.797	4.881	0.027	5.814	1.220 ~ 27.716
Duties (none)	0.270	0.804	0.113	0.736	1.311	0.271 ~ 6.666
C2 compared to C3^(2)^						
(A person’s) age	−0.073	0.065	1.264	0.261	0.930	0.819 ~ 1.056
Years of experience	0.034	0.058	0.350	0.554	1.035	0.923 ~ 1.160
Perceived social support	−0.083	0.018	21.843	<0.001	0.921	0.889 ~ 0.953
Occupational coping self-efficacy	−0.185	0.037	25.468	<0.001	0.831	0.773 ~ 0.893
Marital status	0.388	0.421	0.849	0.357	1.474	0.646 ~ 3.363
Form of employment (establishment)	−0.160	0.421	0.145	0.704	0.852	0.373 ~ 1.945
Duties (none)	−0.840	0.442	3.617	0.057	0.432	0.182 ~ 1.026

C1 is the low job performance type, C2 is the medium job performance type, and C3 is the high job performance type. (1) C1 is used as the reference group; (2) C3 is used as the reference group.

## 4 Discussion

### 4.1 Nurses’ job performance is at a medium-high level

The results of this survey show that nurses’ job performance score is (45.05 ± 6.55), which is at a medium-high level compared to the middle score of 33 on the scale, higher than the results of Song et al.’s study, which may be related to the difference in the subjects of the survey (Song et al., 2024). Song et al. (2024) study focused on young nurses, and Zhao et al.’s study showed that young nurses, who have only been working for a short period of time and are still in the exploratory phase of their clinical careers, may not be able to deal effectively with the complexity and variability of their clinical work due to differences in work experience, skill proficiency, and occupational adaptability, resulting in performance that may not yet have reached a higher level (Zhao et al., 2024). In contrast, this survey covered the entire age group of nurses, and 87.4% of the nurses had more than 5 years of working experience, which showed higher levels of performance due to the valuable experience they had gained over many years of work practice and their skill in communicating with patients and coworkers in order to work together to meet the various nursing challenges and complete the set work tasks.

### 4.2 Nurses’ job performance can be categorized into 3 latent profile analysis

This study identified 3 different nurse job performance categories through LPA, namely “low job performance,” “medium job performance,” “high job performance,” and “high job performance,” which proved the heterogeneity of nurses’ job performance, accounting for 13.9, 52.8, and 33.3%, respectively, “proving the existence of heterogeneity in nurses’ job performance with 13.9, 52.8, and 33.3%, respectively.” Low-job performance nurses” are mainly characterized by low age group, junior title, low working years, and no position. The younger the age of the nurses just entered the workplace, in the early stage of career, this stage of the nurses have solid theoretical knowledge, but lack of corresponding clinical work experience, in the face of complex and changing clinical work, it is often difficult to quickly make accurate judgments and responses, thus affecting the work performance; while the junior title and the low years of experience of the nurses are also faced with the challenge of the transition from theoretical knowledge to The nurses with junior title and low working experience also face the challenge of transitioning from theoretical knowledge to clinical practice. Due to the lack of certain working experience, it is still difficult to adapt to the rhythm of clinical work, and at the same time, they are not good at communicating effectively with colleagues and patients in dealing with emergencies, so they show lower performance; in addition, compared with the head nurse, the nurses with no position not only lack theoretical knowledge and clinical experience, but also find it difficult to compare with the head nurse in terms of management ability and communication skills, so the value of their work is also relatively lower. In addition, the value of the work produced by nurses without positions is relatively low compared to that of nurse managers. Managers should focus on the above characteristics of nurses to carry out targeted training to improve their professional skills and clinical ability to help them quickly adapt to the clinical work environment and role change, so that they can efficiently complete their work tasks. “Medium-performance nurses” are the largest part of the sample, mainly characterized by bachelor’s degree, 26–40 years old, married, and 6–20 years of working experience. These nurses have relatively longer working years, higher education, and married nurses have relatively stable family life. They are able to focus on work-life balance and cherish their career development opportunities, and they have more positive performance in interpersonal interactions and are willing to devote themselves to their work, but the degree of their motivation needs to be further improved. Managers should have in-depth communication with such nurses, and can formulate personalized career planning programs to clarify their career goals and development paths. Through the establishment of title promotion, performance bonuses and other incentives to stimulate their work enthusiasm and creativity, to promote their career development, so that they can be fully engaged in their work, to create a higher level of performance. “High-performance nurses are mainly characterized by their long age, high working experience, married status and high monthly income. These nurses work for a relatively long time and have rich clinical work experience, which can effectively deal with difficulties at work; at the same time, these nurses have a relatively high income, which makes them have a high degree of satisfaction with their work, and their stable marital status can provide them with effective family support, which can help to alleviate difficulties and pressure at work, so that they are willing to be actively involved in it (Cho and Kim, 2022).

### 4.3 Nurses with more years of experience have higher levels of performance

Logistic regression analysis shows that nurses with more years of experience are more likely to enter the “medium performance category” (C1 vs. C2, *OR* = 1.161, *p* < 0.05), which means that compared to nurses in the low performance category, nurses in the medium performance category have higher levels of performance. That is to say, nurses with more years of working experience have relatively higher job performance compared to nurses with low job performance. This may be due to the fact that nurses with more years of working experience have accumulated a wealth of experience in nursing and are more skillful in dealing with common problems and emergencies. This accumulation of experience helps to enhance their work performance, enabling them to perform moderately difficult work tasks and exhibit relatively high work performance. However, prolonged work stress and repetitive tasks may cause nurses to lose enthusiasm for their work, resulting in decreased motivation and initiative. This change in attitude may lead to insufficient motivation to produce high job performance. It is suggested that managers should pay attention to the influence of working seniority on nurses’ work performance; for nurses with low seniority, they should care about their work needs, help them to solve the problems in clinical work and give them full trust and recognition; for nurses with high seniority, they should have a full understanding of their inner true feelings toward their work and the hospital and mobilize their work enthusiasm and motivation so that they can actively engage in their work.

### 4.4 Contract nurses have a higher level of performance

Logistic regression analysis shows that contract nurses are more likely to enter the “medium job performance type” and “high job performance type” (C1 vs. C2, *OR* = 4.954, *p* < 0.05; C1 vs. C3, *OR* = 5.814, *p* < 0.05), which means that contract nurses have relatively higher performance compared to low performance nurses. This may be due to the fact that contract nurses belong to the contractual type, which has relatively poorer job stability compared to the on-staff nurses, making contract nurses cherish their work opportunities in the hospital more, and also hope to obtain more opportunities for promotion as well as development through their own positive work attitudes, and thus show a more proactive work attitude (Whiting et al., 2011). At the same time, the fixed salary and benefits of contract nurses are often worse than those of staffed nurses, making it necessary for them to devote themselves to their work with a high sense of responsibility and positive attitude to ensure their efficient work quality and work efficiency, thus creating more job performance and thus increasing their own remuneration packages. In addition, contract nurses are more fiercely competitive, and they need to manage their time and energy well to ensure that they remain productive and focused in both work and life. This balance will help them to remain productive and efficient, thus showing higher performance. It is suggested that managers should pay attention to the inner motivation of the nurses in the program, and can make career planning together with them, so that they can have a clear cognition of their own development, which can promote the enhancement of their work motivation, and then create more job performance output.

### 4.5 Nurses who comprehend the higher level of social support have higher levels of job performance

Logistic regression analysis showed that nurses who comprehend the higher level of social support are more likely to enter the “medium job performance” and “high job performance” (C1 vs. C2, *OR* = 1.060, *p* < 0.05; C1 vs. C3, *OR* = 1.152, *p* < 0.05; C3 vs. C2, *OR* = 0.921, *p* < 0.05), which means that nurses with higher perceived social support have higher job performance compared to nurses with low job performance. This may be due to the fact that when nurses perceive more support from the organization, family, and society, they are able to effectively reduce their own work pressure and have a high sense of recognition for their profession, thus willing to give more time and energy for their work (Xu et al., 2020). Meanwhile, according to the social exchange theory proposed that social support triggers social exchange, when nurses perceive more caring support among leaders and coworkers, they will show a positive state of work engagement at work, and at the same time be able to create more work output (Wan et al., 2018). It is suggested that managers (1) should pay attention to nurses with low levels of perceived social support and actively create a supportive work atmosphere so that they can perceive care and support from the organization and among colleagues; (2) actively mobilize family members to support and care for nurses to reduce the conflict between work and family, so that they can be positively engaged in their work.

### 4.6 Nurses with higher occupational coping self-efficacy have higher levels of job performance

Logistic regression analysis showed that nurses with higher occupational coping self-efficacy were more likely to enter the “moderate job performance” and “high job performance” (C1 vs. C2, *OR* = 1.099, *p* < 0.05; C1 vs. C3, *OR* = 1.322, *p* < 0.05; C3 vs. C2, *OR* = 0.831, *p* < 0.05), which indicates that nurses with higher self-efficacy for occupational coping have higher job performance compared with nurses with low job performance. Resource conservation theory suggests that the psychological capital possessed by an individual is the core psychological factor that promotes his or her own development and enhances job performance, and self-efficacy, as an important part of an individual’s psychological capital, has a direct positive effect on job performance (Bai et al., 2021). When nurses have a higher level of occupational coping self-efficacy, they will show good self-confidence and are good at mobilizing positive psychological resources to solve the dilemmas encountered in the work, so as to efficiently complete the work tasks and improve work performance. Tips for managers: (1) To pay attention to nurses with low self-efficacy in occupational coping, we can start from carrying out positive psychological training courses, reduce nurses’ work pressure through self-regulation and positive thoughts to reduce stress, and enhance their psychological capital, so as to improve their self-confidence and coping ability at work. (2) By mobilizing the exogenous support of nurses so that they can be recognized by society, patients and their families, etc., thus enhancing their inner core motivation at work, and then devote themselves to their work to provide patients with more efficient nursing services.

## 5 Limitations

This study also has some limitations due to time and effort constraints. First, all entries in this study were completed using self-report, which may be affected by recall bias and reporting bias due to individuals’ own conditions; second, this study was cross-sectional and lacked the distribution of a controlled sample, which may lead to nonresponse bias and the strength of causal arguments in cross-sectional studies is not very strong; lastly, only 5 out of the 30 general hospitals were selected for this study, the sample was somewhat underrepresented, and extrapolation of the results may have been hampered. In subsequent studies, the sample size and the scope of sample selection should be expanded, and multi-center, large-sample studies should be conducted to explore the performance of nurses in different regions of China. At the same time, it is necessary to conduct a longitudinal study to explore the changes in the performance of nurses at different points in time, so as to increase the strength of argumentation of the results of the study, and to provide a reference basis for the subsequent development of appropriate interventions to improve the job performance of nurses.

## 6 Contributions

This study has three major contributions. First, we provide empirical evidence on the current status of nurses’ job performance in our region as well as an analysis of heterogeneity, demonstrating significant associations between years of experience, contract type, occupational coping self-efficacy, and perceived social support and job performance, and providing support for policy makers in the region to take measures to improve nurses’ job performance. Second, we emphasized the importance of job performance in improving the quality of nursing services, which provides a direction for nurses’ future career development. Finally, this study not only expands the theoretical model of nursing job performance, but also provides new analytical tools that offer valuable guidance for future research and practice.

## 7 Conclusion

This study further explored that the job performance of nurses in this region is at a medium-to-high level, which still needs to be improved. The study used latent profile analysis to divide the job performance of nurses into three types: “Low job performance Type,” “Medium job performance Type,” and “High job performance Type,” with nurses in the three groups exhibiting group heterogeneity. Nurses with longer years of work experience, contract-based employment, higher occupational coping self-efficacy, and higher perceived social support tend to have higher levels of work performance. Therefore, nursing managers should focus on nurses with “low job performance” and “medium job performance.” For nurses with “low job performance,” it is necessary to work with them to develop career opportunities, clarify short-term and long-term goals, and help them improve professional skills and occupational coping confidence through systematic professional training and stress management courses. At the same time, it is also possible to reduce the workload of nurses through reasonable human resource allocation and enhance their intrinsic motivation by improving salary and benefits, enabling them to efficiently achieve work objectives. For nurses with “medium job performance,” it is necessary to strengthen care and support, help nurses identify areas for improvement, and set reasonable challenges and goals to motivate them to enhance their work performance, so that they can actively engage in their work and provide high-quality nursing services to patients.

## Data Availability

The raw data supporting the conclusions of this article will be made available by the authors, without undue reservation.
